# Validation of an early vascular aging construct model for comprehensive cardiovascular risk assessment using external risk indicators for improved clinical utility: data from the EVasCu study

**DOI:** 10.1186/s12933-023-02104-y

**Published:** 2024-01-13

**Authors:** Iván Cavero-Redondo, Alicia Saz-Lara, Irene Martínez-García, Iris Otero-Luis, Arturo Martínez-Rodrigo

**Affiliations:** 1 Health and Social Research Center, University of Castilla-La Mancha, Cuenca, Spain; 2 Facultad de Ciencias de la Salud, Universidad Autónoma de Chile, Talca, Chile; 3 Research Group in Electronic, Biomedical, and Telecommunication Engineering, University of Castilla-La Mancha, Cuenca, Spain

**Keywords:** Cardiovascular Diseases, Early vascular aging, Risk assessment, Clustering model, Cardiovascular risk factors.

## Abstract

**Background:**

Cardiovascular diseases (CVDs) remain a major global health concern, necessitating advanced risk assessment beyond traditional factors. Early vascular aging (EVA), characterized by accelerated vascular changes, has gained importance in cardiovascular risk assessment.

**Methods:**

The EVasCu study in Spain examined 390 healthy participants using noninvasive measurements. A construct of four variables (Pulse Pressure, Pulse Wave Velocity, Glycated Hemoglobin, Advanced Glycation End Products) was used for clustering. K-means clustering with principal component analysis revealed two clusters, healthy vascular aging (HVA) and early vascular aging (EVA). External validation variables included sociodemographic, adiposity, glycemic, inflammatory, lipid profile, vascular, and blood pressure factors.

**Results:**

EVA cluster participants were older and exhibited higher adiposity, poorer glycemic control, dyslipidemia, altered vascular properties, and higher blood pressure. Significant differences were observed for age, smoking status, body mass index, waist circumference, fat percentage, glucose, insulin, C-reactive protein, diabetes prevalence, lipid profiles, arterial stiffness, and blood pressure levels. These findings demonstrate the association between traditional cardiovascular risk factors and EVA.

**Conclusions:**

This study validates a clustering model for EVA and highlights its association with established risk factors. EVA assessment can be integrated into clinical practice, allowing early intervention and personalized cardiovascular risk management.

## Introduction

Currently, cardiovascular diseases (CVDs) continue to be a leading cause of morbidity and mortality on a global scale, presenting an ongoing challenge to healthcare systems and medical research communities [1]. Traditionally, CVD risk has been evaluated by using conventional methods focused on established risk factors, such as hypertension, elevated cholesterol levels, diabetes, or smoking habits [2]. While these traditional parameters have provided valuable insights, they have been shown to be insufficient in accounting for the actual incidence of CVD events [3]. Specifically, the number of individuals who ultimately experience cardiovascular events often exceeds those predicted to be at high risk, particularly among populations over the age of 40 [4]. This incongruence, commonly referred to as the “detection gap,” raises questions about the completeness of current risk assessment models and points out the need for additional predictive variables that go beyond these classical factors [5].

Beyond its role as a detection bridge in cardiovascular risk assessment, Early Vascular Aging (EVA) presents a multifaceted challenge, unraveling the intricate complexity of its underlying mechanisms [6]. The accelerated vascular changes observed in EVA, such as arterial stiffening and endothelial dysfunction, pose a complex puzzle for researchers and clinicians alike [7]. Understanding the nuanced interplay of these structural and functional alterations is crucial not only for accurate risk assessment but also for devising targeted interventions to impede or reverse the progression of vascular aging [8]. Unraveling the molecular and cellular pathways involved in EVA remains an ongoing pursuit, holding the key to unlocking novel therapeutic avenues in the battle against cardiovascular diseases [6].

EVA has increasingly been recognized as a key factor that could fill the detection gap inherent in traditional cardiovascular risk assessment models [9]. EVA manifests through a collection of both structural and functional alterations in the vascular system. Such changes include arterial stiffness, endothelial dysfunction, and variations in the intima-media thickness of the vessel wall [10]. Moreover, these vascular alterations, while associated with normal ageing, occur at an accelerated rate in EVA, culminating in decreased arterial distensibility and increased arterial stiffness. Consequently, accelerated vascular aging contributes to a number of cardiovascular complications, most notably related to coronary artery disease and stroke [11]. Therefore, the quantification of EVA risk has become a key factor in the strategic prevention and management of cardiovascular diseases [12].

As we delve into the realm of EVA assessment, a paradigm shift towards unsupervised learning techniques unfolds, which can reveal hidden patterns and relationships within vascular risk [13]. Unlike traditional classification methods, which predominantly focus on individuals with documented cardiovascular events, unsupervised techniques cover latent risks that may not manifest explicit symptoms [14]. This shift, while promising, is not without its challenges, particularly the absence of a universally accepted gold standard for EVA prediction. Consequently, the validation of unsupervised models becomes paramount, prompting innovative approaches such as comparing emergent risk patterns with external variables like age, body fat percentage, or cholesterol levels. Navigating these uncharted territories in EVA assessment holds the potential to revolutionize risk prediction models and enhance our understanding of vascular aging dynamics [15].

Traditionally, vascular risk assessment using EVA has been approached through classification techniques [16]. While these methods have provided valuable insights, their primary focus has been on patients who have either manifested a cardiovascular event or for whom such an event has been documented. However, this methodology might inadvertently omit individuals exhibiting latent risk who have yet to display explicit symptoms or have an event recorded [12]. For this reason, a shift towards unsupervised learning techniques in EVA assessment could uncover underlying risk patterns and identify novel and potentially overlooked relationships between risk factors. However, the absence of a recognized gold standard for EVA prediction presents a significant challenge, emphasizing the need for validating the risk models developed through unsupervised techniques [9]. Given this context, one promising approach for validation involves employing a set of external variables not initially included in the unsupervised model [17]. Specifically, comparing the unsupervised groups against external features, recognized as indicators of premature vascular risk, such as age, body fat percentage or cholesterol, could serve as valuable benchmarks to assess and corroborate the reliability and robustness of these emergent risk models.

In recent research, a construct composed of four variables consisting of pulse pressure (PP), pulse wave velocity (PWv), glycated hemoglobin (HbA1c), and advanced glycation end products (AGEs) measured by skin autofluorescence (SAF) was introduced by our research group as a potential tool for assessing vascular risk [9]. Preliminary analyses of this construct indicated that optimal differentiation into two clusters provided the most coherent grouping of individuals regarding the risk of suffering from EVA. The selection of these variables is based on an understanding of the physiological changes associated with EVA that goes beyond classical risk factors. This comprehensive approach addresses both the structure and function of the vascular system, providing a more complete picture of the mechanisms underlying cardiovascular risk [18]. PP serves as an indicator of the force exerted by the blood on the arterial walls during each heartbeat [19], PWv reflects arterial stiffness [20], HbA1c serves as an indicator of long-term glycaemic control that impacts vascular health [21], and SAF indicate accumulated tissue damage [22].

While this construct offers a novel perspective, it remains essential to further validate its effectiveness. By doing so, we can not only potentially highlight the inherent relationships and patterns within the data but also confirm the robustness and validity of both the construct and the clustering model. Therefore, the objective of this work is to validate the proposed clustering model by comparing its outcomes with external vascular risk indicators, ensuring a comprehensive and practical understanding of EVA. Successful validation of this approach could facilitate its integration into clinical settings, providing healthcare professionals with a robust tool for early and accurate risk assessment.

## Materials and methods

### Study design

The EVasCu study was a cross-sectional investigation conducted in the province of Cuenca, Spain, to evaluate the validity of an early vascular aging model as an index of cardiovascular risk in healthy adults [9]. The study adhered to the principles outlined in the Declaration of Helsinki and received prior approval from the Clinical Research Ethics Committee of the Cuenca Health Area (REG: 2022/PI2022). The study design followed the guidelines provided by the Strengthening the Reporting of Observational Studies in Epidemiology (STROBE) statement [23].

### Study sample

A total of 390 participants from Cuenca (Spain) were recruited for the EVasCu study between June 2022 and December 2022. Individuals with preexisting conditions that could significantly influence arterial stiffness, including diabetes mellitus, dyslipidemia, and arterial hypertension, were excluded from the study. Participants were provided with detailed information about the purpose and procedures of the study, and written informed consent was obtained. After the data collection, participants received validated reports from a physician, along with appropriate recommendations if needed.

### EVA construct variables

PWv was measured using oscillometric techniques with Mobil-O-Graph® (IEM GmbH). Mobil-O-Graph® measures aortic pulse wave velocity (PWv) calculated as the mean of two repeated measurements, separated by 5 min each. These parameters were measured in a quiet place and after a 5-min rest period using a cuff size according to the participant’s arm/s and/or lower limb circumference.

Pulse pressure (PP) was obtained from the difference between mean systolic blood pressure (SBP) and diastolic blood pressure (DBP). Blood pressure was measured in a quiet place and after a 5-min rest period using the Omron® M5-I monitor (Omron Healthcare UK Ltd.) with a cuff size according to the participant’s arm circumference.

Glycated hemoglobin A1c (HbA1c) was determined by high-performance liquid chromatography using the ADAMS A1c HA-8180 V analyser from A. Menarini Diagnostics®. Samples were collected between 8 a.m. and 9 a.m. and after 12 h of fasting.

AGEs were measured by SAF with the AGE Reader® device. SAF were calculated as the mean of the measurements from both arms. The mean for each arm was calculated as the mean of three repeated measurements.

### External validation variables

#### Sociodemographic and lifestyle factors

Age, gender, and smoking status were collected through direct questioning. Smoking status was classified into five groups: smoker, ex-smoker < 1 year, ex-smoker 1–5 years, ex-smoker > 5 years, and nonsmoker.

#### Adiposity factors

Weight and height were measured twice using appropriate equipment and averaged for analysis. Body mass index (BMI) was calculated as weight in kilograms divided by the squared height in meters (kg/m2), and participants were classified as underweight, normal weight, overweight, or obese using cut-off points of 18.5, 25.0, and 30.0, respectively [24]. Fat percentage was measured by calculating the average of two measurements using Tanita® BC-418 MA 8-electrode electrical bioimpedance.

#### Glycemic and inflammatory factors

Glucose and ultrasensitive C-reactive protein (CRP) determinations were measured on a Roche Diagnostics® Cobas 8000 system, and insulin determinations were measured on the Abbott ® Architect platform. Samples were collected between 8 a.m. and 9 a.m. and after 12 h of fasting. Diabetic status was established according to the HbA1c criteria for the diagnosis of diabetes of the American Diabetes Association (ADA) [25]: nondiabetic (HbA1c < 5.7%), prediabetic (HbA1c 5.7–6.4%) and diabetic (HbA1c ≥6.5%).

#### Lipid profile factors

Total cholesterol, low-density lipoprotein cholesterol (LDL), high-density lipoprotein cholesterol (HDL) and triglyceride determinations were measured on a Roche Diagnostics® Cobas 8000 system, and insulin determinations were obtained on the Abbott ® Architect platform. Samples were collected between 8 a.m. and 9 a.m. and after 12 h of fasting. Hypercholesterolemia and hypertriglyceridaemia status were established according to the total cholesterol and triglyceride criteria of the American Heart Association (AHA) and the American College of Cardiology (ACC) [26], considering total cholesterol > 200 mg/dL for hypercholesterolemia and triglycerides > 150 mg/dL for hypertriglyceridaemia.

#### Vascular factors

The augmentation index (AIx75) was measured using Mobil-O-Graph®, which was calculated as the mean of two repeated measurements, separated by 5 min each. Cardio-ankle vascular index (CAVI) and ankle-brachial index (ABI) were measured with the VaSera system VS-1500 N (Fukuda Denshi UK Ltd.). Finally, intima-media thickness (IMT) was measured by ultrasound with the Sonosite SII device (Sonosite Inc., Bothell, Washington, USA). IMT was calculated as the mean measurement of the right and left carotid arteries.

#### Blood pressure factors

Blood pressure was measured in a quiet place and after a 5-min rest period using the Omron® M5-I monitor (Omron Healthcare UK Ltd. with a cuff size according to the participant’s arm circumference. SBP and DBP were calculated as the mean of two repeated measurements, separated by 5 min each. Hypertension status was established according to the SBP and DBP criteria of the AHA and ACC [26]: Optima blood pressure (SBP < 120 mmHg and/or DBP < 80 mmHg), normal blood pressure (SBP 120–129 mmHg and/or DBP 80–84 mmHg), normal-high blood pressure (SBP 130–139 mmHg and/or DBP 85–89 mmHg), hypertension grade I (SBP 140–159 mmHg and/or DBP 90–99 mmHg), hypertension grade II (SBP ≥160 mmHg and/or DBP ≥100 mmHg) and hypertension grade III (SBP ≥180 mmHg and/or DBP ≥110 mmHg).

It should be clarified that all subjects included in the EVasCu study were healthy subjects, so diabetic, hypercholesterolemia, hypertriglyceridaemia and hypertension status were established on the basis of the guidelines mentioned above and would not be subjects diagnosed with these diseases at the time of data collection, so they were not treated for these diseases and were informed with a report to be diagnosed by a physician.

### Statistical analysis

#### Data description and preprocessing

Before any analyses, a preprocessing stage was conducted on the dataset. First, when features presented missing values, they were imputed using their respective medians for each feature. Second, to ensure a consistent scale across variables, all features were standardized using z score normalization.

#### Cluster formation and data representation

Initially, the optimal number of risk groups, denoted as K, was determined based on the inherent characteristics of the four-variable construct. Different values of K (ranging from 2 to 5) were tested using the Calinski‒Harabasz, Davies‒Bouldin, and Silhouette indices to evaluate the quality and coherence of the formed clusters [27, 28]. These indices were also crucial to determine the cohesion and separation within and between the clusters once they were computed, thus measuring the quality of the grouping [29].

Later, patients were assigned to these groups using the K-means algorithm [30]. Mathematically, this algorithm minimizes the sum of squared Euclidean distances between each point and its assigned centroid. In this process, each patient was allocated to the nearest group or centroid based on the shortest Euclidean distance. This assignment process was rigorously iterated until the centroid positions experienced negligible changes or until a maximum of 100 iterations was reached. To further elucidate the structure of the data and to visualize the delineation between clusters, principal component analysis (PCA) was utilized, especially emphasizing the distinction between clusters derived from the original four-variable construct.

#### External validation analysis

To assess the distribution of external variables within each cluster, Shapiro-Wilks and Levene’s tests were employed, which determine the normality and homoscedasticity of the data, respectively. Based on the outcomes of these preliminary tests, the appropriate statistical analysis to discern differences between clusters was chosen. Thus, when a quantitative variable exhibited a normal distribution and homogenous variances across both clusters, Student’s t test was applied. Conversely, when the assumptions of normality or homoscedasticity were not met, the nonparametric Mann‒Whitney U test was chosen. For categorical variables, the chi-square test was used. These tests ensure robust identification of significant differences between clusters based on the data characteristics.

Clustering modelling was conducted using Python version 3.10 and the scikit-learn package [31], whereas statistical analyses were performed with SPSS version 28.

## Results

### Characteristics of study participants

The EVasCu study sample included a total of 390 participants, of whom 246 (63.1%) were women. The mean age of the participants was 42.0 ± 13.1 years. Table 1 shows the baseline characteristics of the enrolled population.

**Table 1:** Characteristics of the participants in the EVasCu study

	Total	HVA	EVA	*p*-value
Sample (n)	390	232	158	
Age, years	42.1 (13.2)	34.9 (10.9)	52.5 (8.2)	< 0.001
Gender, %				
Women	63.1	64.2	61.4	0.549
Men	36.9	35.8	38.6	
Smoking status, %				
Nonsmoker	63.6	72.8	50.0	< 0.001
Ex-smoker (> 5 years)	18.7	11.2	29.7	
Ex-smoker (1–5 years)	3.3	0.9	7.0	
Ex-smoker (0–1 year)	1.8	1.7	1.9	
Current smoker	12.6	13.4	11.4	
Weight, kg	70.1 (14.3)	67.9 (13.6)	73.7 (15.3)	< 0.001
Height, m	1.7 (0.1)	1.7 (0.1)	1.7 (0.1)	0.197
BMI, kg/m^2^	24.8 (4.2)	23.9 (3.7)	26.3 (4.5)	< 0.001
Waist circumference, cm	82.7 (12.8)	79.1 (10.9)	87.9 (13.7)	< 0.001
Fat percentage. %	27.2 (9.4)	25.5 (8.9)	29.8 (9.5)	< 0.001
Weight status, %				
Underweight	2.8	3.9	1.2	< 0.001
Normal weight	52.4	60.8	41.1	
Overweight	32.1	27.6	37.3	
Obesity	12.6	7.8	20.3	
PWv, m/s	6.3 (1.4)	5.6 (0.9)	7.5 (1.1)	< 0.001
CAVI, m/s	7.1 (1.2)	6.7 (1.0)	7.6 (1.2)	< 0.001
ABI	1.1 (0.1)	1.1 (0.1)	1.1 (0.1)	0.195
AIx-75, %	16.8 (11.9)	14.8 (11.9)	19.5 (11.6)	< 0.001
IMT, mm	0.2 (0.1)	0.2 (0.1)	0.2 (0.1)	0.009
SBP, mmHg	116.6 (15.2)	112.6 (12.4)	122.7 (16.9)	< 0.001
DBP, mmHg	70.3 (10.6)	68.2 (9.6)	73.5 (11.2)	< 0.001
PP,mmHg	46.3 (9.9)	44.4 (9.3)	48.8 (10.8)	< 0.001
Hypertension status, %				
Optima	61.1	71.4	45.9	< 0.001
Normal	19.6	17.7	22.2	
Normal-High	9.8	6.9	13.9	
Hypertension Grade I	7.5	3.0	13.9	
Hypertension Grade II	1.8	0.9	3.2	
Hypertension Grade III	0.3	0.0	0.6	
HbA1c, %	5.2 (0.3)	5.0 (0.3)	5.4 (0.3)	< 0.001
Glucose, mg/dL	89.4 (9.9)	86.3 (8.4)	94.0 (10.3)	< 0.001
Insulin, mg/dL	8.5 (6.1)	7.9 (5.7)	9.5 (6.6)	0.04
Diabetes status, %				
Nondiabetic	98.4	100	96.2	0.012
Prediabetic	1.3	0.0	3.2	
Diabetic	0.3	0.0	0.6	
SAF, au	1.9 (0.4)	1.7 (0.3)	2.2 (0.4)	< 0.001
CRP, mg/L	1.8 (4.1)	1.9 (4.7)	1.6 (2.9)	0.600
Total cholesterol, mg/dL	187.6 (36.2)	179.3 (35.0)	199.8 (34.6)	< 0.001
HDL, mg/dL	61.5 (13.7)	62.0 (13.8)	60.8 (13.8)	0.392
LDL, mg/dL	118.9 (32.9)	110.9 (31.0)	130.6 (32.3)	< 0.001
Triglycerides, mg/dL	87.2 (48.8)	80.7 (43.7)	96.9 (54.1)	0.002
Hypercholesterolemia status, %				
Non-hypercholesterolemia	64.9	73.3	52.5	< 0.001
Hypercholesterolemia	35.1	26.7	47.5	
Hypertriglyceremia status, %				
Non-hypertriglyceridaemia	90.3	92.7	86.7	0.051
Hypertriglyceridaemia	9.7	7.3	13.3	

Data are shown as the mean (SD) (continuous variables) and percentage (categorical variables). HVA, healthy vascular aging; EVA, early vascular aging; BMI, body mass index; PWv, pulse wave velocity; CAVI, cardio-ankle vascular index; ABI, ankle-brachial index; AIx@75, augmentation index; IMT: intima media thickness; SBP, systolic blood pressure; DBP, diastolic blood pressure; PP, pulse pressure; HbA1c: glycated hemoglobin A1c; SAF: advanced glycation products measured by skin autofluorescence; CRP, c-reactive protein; HDL, high-density lipoprotein cholesterol; LDL, low-density lipoprotein cholesterol

#### Cluster analysis

**Fig. 1:**
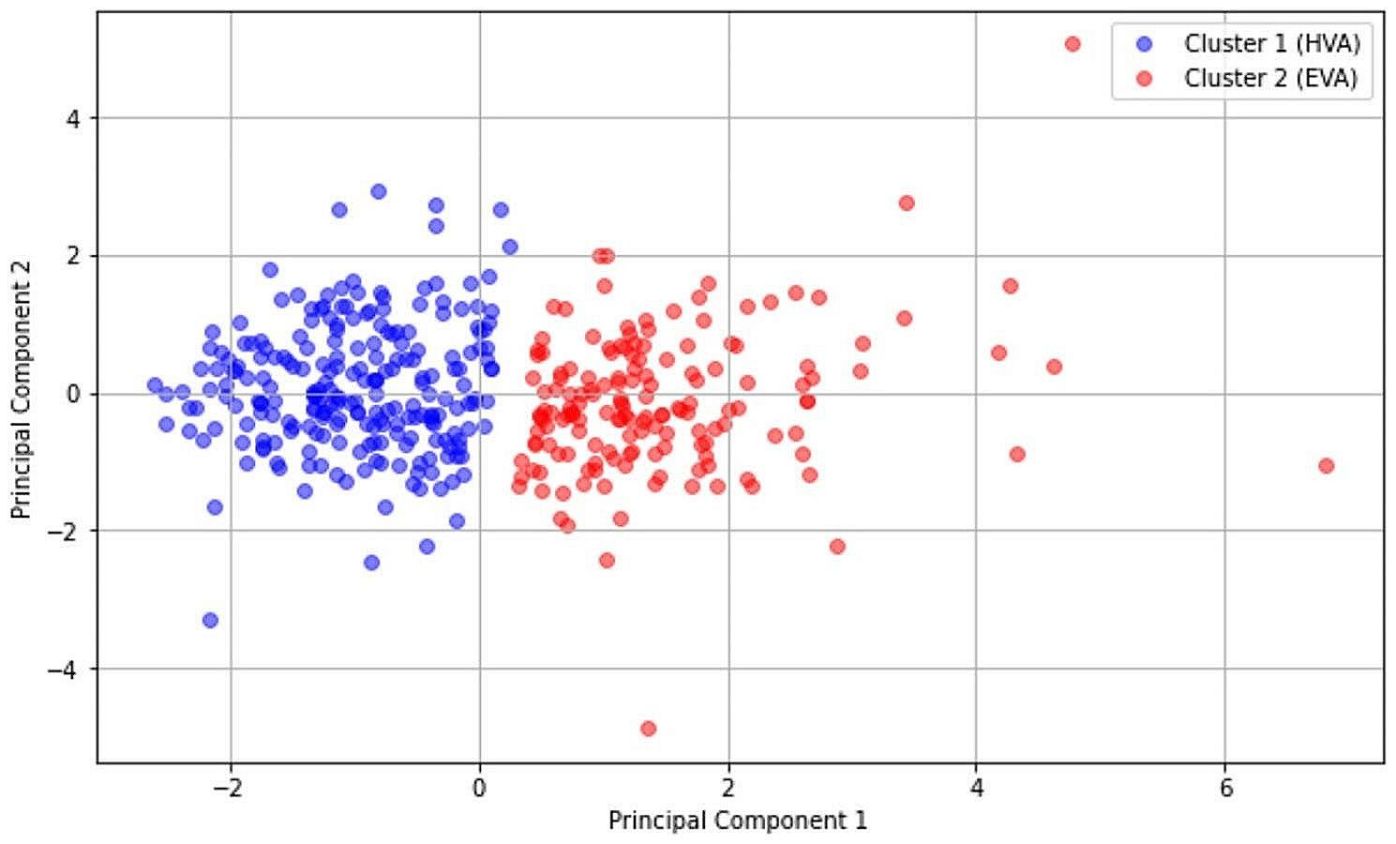
2D PCA view with K-means cluster

This study is constructed upon a clustering model previously established in preceding research [6]. In that study, the robustness and coherence of the clustering groups were rigorously demonstrated, showcasing the model’s reliability [6]. Building on this solid foundation, the following analyses seek to further validate the clustering, with the objective of affirming its applicability and consistency across practical applications. Figure 1 shows a 2-D visual representation of clusters using the first two principal components with normalized data, which explains more than 75% of the total data variability of the construct variables. Notably, the most robust model configuration was identified when utilizing two distinct groups.

### Identifying health and risk groups in the cluster model

**Fig. 2:**
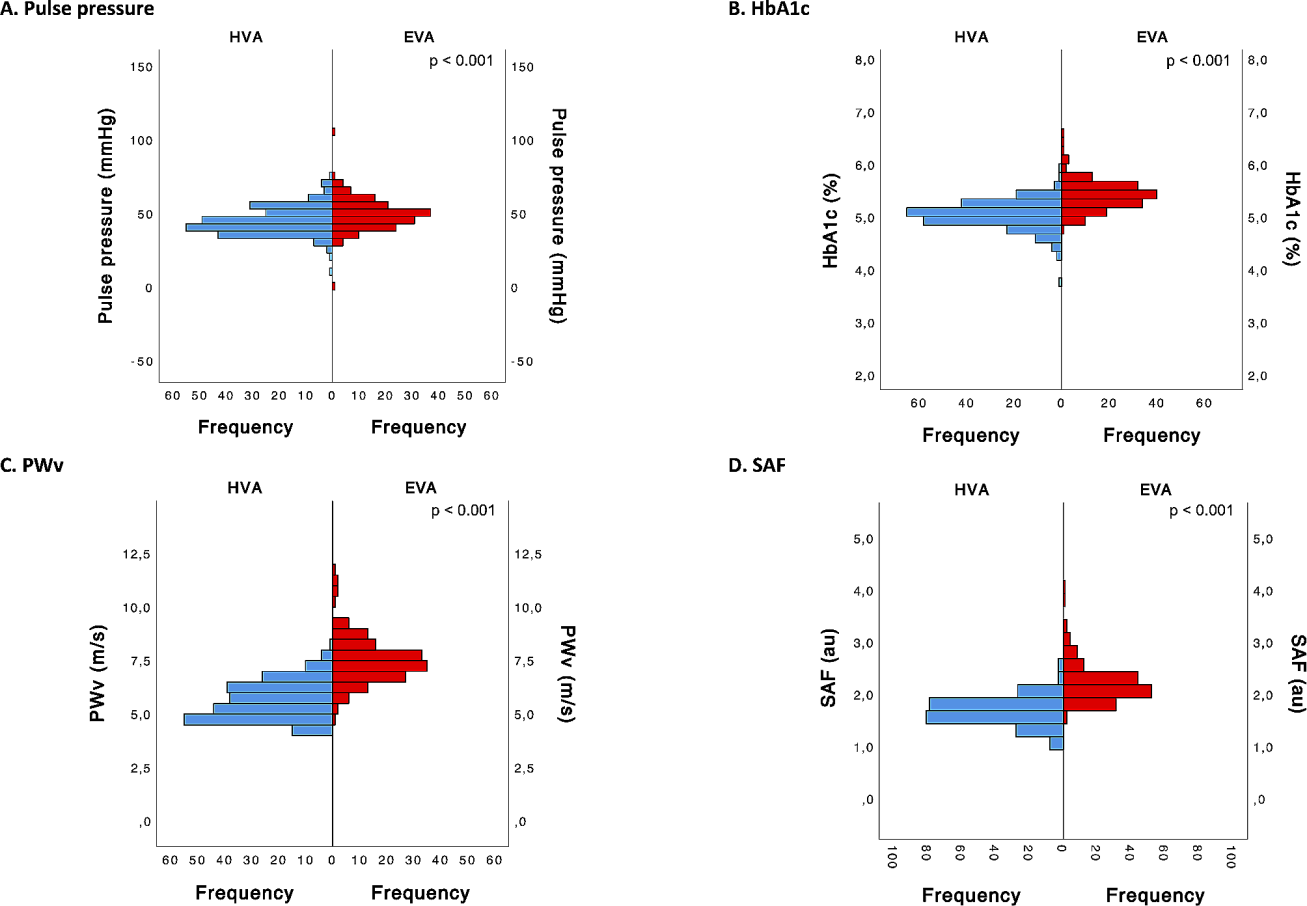
Differences between the cluster categories (HVA and EVA) for sociodemographic and lifestyle factors For **A**, **B**, **C** and **D**, *p* values were estimated using the Mann‒Whitney U test. HVA, healthy vascular aging; EVA, early vascular aging; HbA1c: glycated hemoglobin A1c; PWv, pulse wave velocity; SAF: advanced glycation products measured by skin autofluorescence

Table 2 presents the definitions for the two clusters identified within the EVasCu study, referred to as Cluster 1 and Cluster 2 (see Fig. 1), based on the selected construct variables. The median values and interquartile ranges (IQRs) for each variable are presented, and *p* values are provided to highlight any statistically significant differences between the two clusters. The data in Table 2, complemented by the visual representation in Fig. 2, reveal marked disparities between the two clusters across all the construct variables. Indeed, Cluster 2 exhibited higher values of pulse pressure, HbA1c, PWv, and SAF than Cluster 1, with these differences achieving statistical significance (*p* < 0.001). Consequently, Cluster 1 can be confidently associated with the HVA, while Cluster 2 can be associated with the EVA. In subsequent analyses, this grouping aims to be further validated, employing external variables not used in the initial construct of the model, thus ensuring the robustness and applicability of this assignment.

**Table 2:** Differences between the cluster categories (HVA and EVA) in the variables included in the EVA construct model

	HVA		EVA		*p* value
Construct variable	Median	IQR	Median	IQR	
Pulse pressure, mmHg	43,0	38.0–50.0	48.0	42.5–55.5	< 0.001
HbA1c, %	5.1	4.9–5.2	5.4	5.2–5.6	< 0.001
PWv, m/s	5.5	4.8–6.3	7.4	6.7-8.0	< 0.001
SAF, au	1.7	1.5–1.8	2.2	2.0-2.4	< 0.001

HVA, healthy vascular aging; EVA, early vascular aging; IQR: interquartile range; HbA1c: glycated hemoglobin A1c; PWv, pulse wave velocity; SAF: advanced glycation products measured by skin autofluorescence

#### Quantitative and categorical risk indicators

Tables 3 and 4 provide a comprehensive analysis of quantitative and categorical risk indicators, categorized into distinct factors, enabling an in-depth exploration of the multifaceted risk factors present within the EVasCu study.

**Table 3:** Differences between the cluster categories (HVA and EVA) in the quantitative risk factors

Variables	HVA		EVA		*p* value
	Median	IQR	Median	IQR	
*Sociodemographic factors*					
Age, years	35.0	24.3–44.0	53.0	48.0–58.0	< 0.001
Adiposity factors					
BMI, kg/m2	23.6	21.3–23.8	25.9	23.3–29.3	< 0.001
Waist circumference, cm	78.4	70.3–86.2	87.2	79.3–96.9	< 0.001
Fat percentage. %	24.9	19.2–31.8	29.9	23.9–37.5	< 0.001
*Glycemic and inflammatory factors*					
Glucose, mg/dL	87.0	81.0-90.8	93.0	86.0–99.0	< 0.001
Insulin, mg/dL	6.6	4.5–9.4	7.1	4.8–11.6	0.034
CRP, mg/L	0.8	0.4–1.5	1.0	0.5–1.5	0.031
*Lipid profile factors*					
Total cholesterol, mg/dL	176.0	152.3-202.7	197.5	176.5-223.3	< 0.001
HDL, mg/dL	61.5	52.0–70.0	61.0	50.5–69.1	0.498
LDL, mg/dL	108.0	87.0-132.0	127.0	109.0-152.0	< 0.001
Triglycerides, mg/dL	68.5	56.0–91.0	81.5	63.8–114.0	< 0.001
*Vascular factors*					
AIx-75, %	16.5	5.0–24.0	19.0	10.0–29.0	< 0.001
ABI	1.1	1.1–1.2	1.1	1.1–1.2	0.115
CAVI, m/s	6.8	6.1–7.3	7.4	7.0-8.4	< 0.001
IMT, mm	0.2	0.1–0.3	0.2	0.2–0.3	0.015
*Blood pressure factors*					
SBP, mmHg	111.0	104.0-120-0	122.0	110.0-134.5	< 0.001
DBP, mmHg	68.0	62.0–74.0	73.0	65.0–82.0	< 0.001

HVA, healthy vascular aging; EVA, early vascular aging; IQR: interquartile range; BMI, body mass index; CRP, c-reactive protein; HDL, high-density lipoprotein cholesterol; LDL, low-density lipoprotein cholesterol; AIx@75, augmentation index; ABI, ankle-brachial index; CAVI, cardio-ankle vascular index; IMT: intima media thickness; SBP, systolic blood pressure; DBP, diastolic blood pressure

**Table 4:** Differences between the cluster categories (HVA and EVA) in the categorical risk factors

	HVA	EVA	*p* value
	n (%)	n (%)	
*Sociodemographic factors*			
Gender			0.549
Women	149 (64.2)	97 (61.4)	
Men	83 (35.8)	61 (38.6)	
Smoking status			< 0.001
Nonsmoker	169 (72.8)	79 (50.0)	
Ex-smoker (> 5 years)	26 (11.2)	47 (29.7)	
Ex-smoker (1–5 years)	2 (0.9)	11 (7.0)	
Ex-smoker (0–1 year)	4 (1.7)	3 (1.9)	
Current smoker	31 (13.4)	18 (11.4)	
*Adiposity factors*			
Weight status			< 0.001
Underweight	9 (3.9)	2 (1.2)	
Norma weight	141 (60.8)	65 (41.1)	
Overweight	64 (27.6)	59 (37.3)	
Obesity	18 (7.8)	32 (20.3)	
*Glycemic factors*			
Diabetes status			0.012
Nondiabetic	232 (100)	152 (96.2)	
Prediabetic	0 (0.0)	5 (3.2)	
Diabetic	0 (0.0)	1 (0.6)	
*Lipid profile factors*			
Hypercholesterolemia status			< 0.001
Non-Hypercholesterolemia	170 (73.3)	83 (52.5)	
Hypercholesterolemia	62 (26.7)	75 (47.5)	
Hypertriglyceremia status			0.051
Non-Hypertriglyceremia	215 (92.7)	137 (86.7)	
Hypertriglyceremia	17 (7.3)	21 (13.3)	
*Bloopd pressure factors*			
Hypertension status			< 0.001
Optima	166 (71.4)	73 (45.9)	
Normal	41 (17.7)	35 (22.2)	
Normal-High	16 (6.9)	22 (13.9)	
Hypertension Grade I	7 (3.0)	22 (13.9)	
Hypertension Grade II	2 (0.9)	5 (3.2)	
Hypertension Grade III	0 (0.0)	1 (0.6)	

HVA, healthy vascular aging; EVA, early vascular aging

**Fig. 3:**
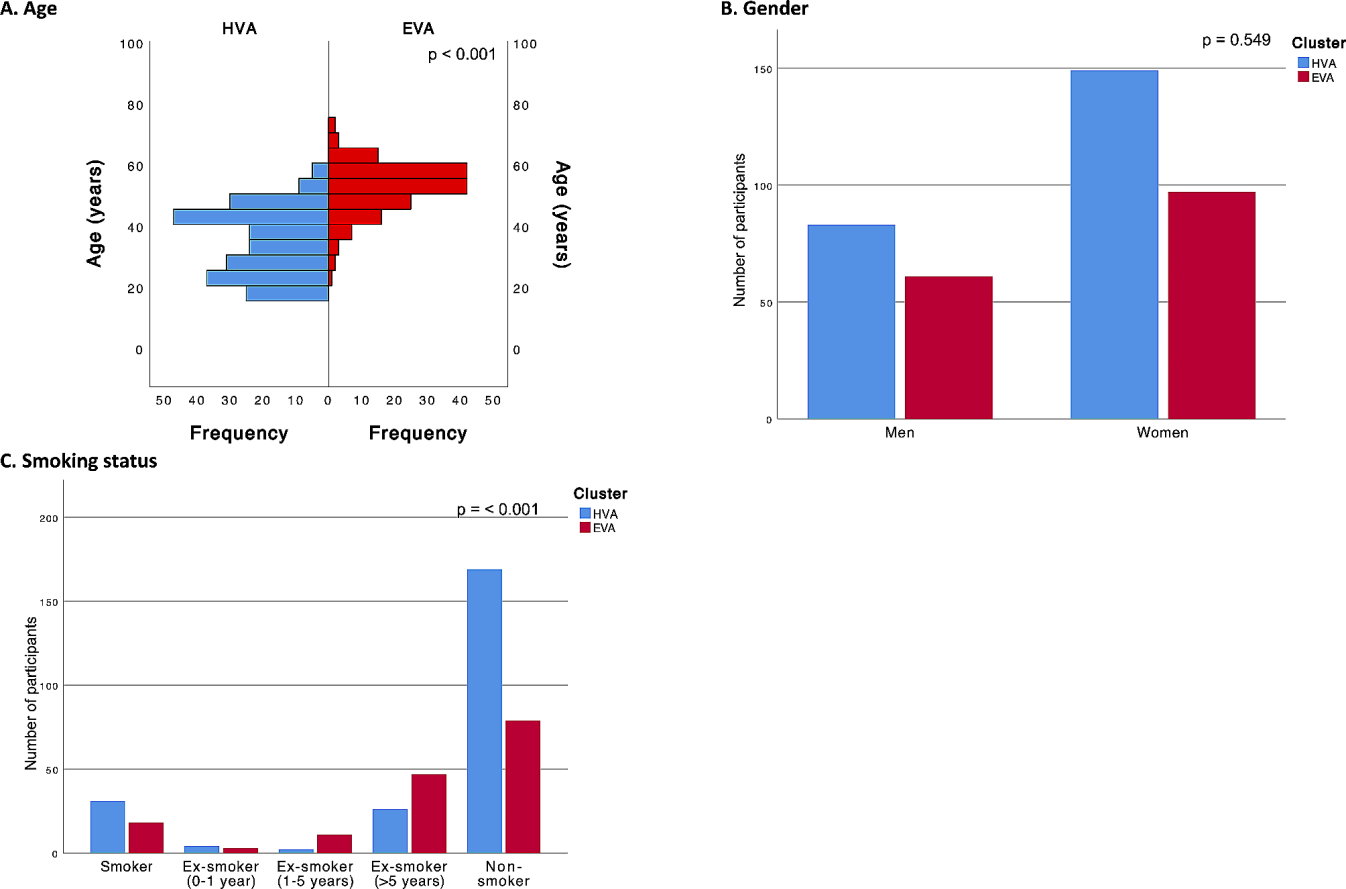
Differences between the cluster categories (HVA and EVA) for sociodemographic and lifestyle factors For **A**, *p* values were estimated using the Mann‒Whitney U test, and for **B** and **C**, *p* values were estimated using the chi-squared test. HVA, healthy vascular aging; EVA, early vascular aging

In terms of sociodemographic and lifestyle factors, age emerged as a substantial differentiator, with individuals in the EVA cluster showing a marked increase in age compared to their HVA counterparts (*p* < 0.001). In contrast, the gender distribution remained statistically homogeneous between the two clusters. However, a notable trend emerged in smoking status, where although similar prevalences were observed in current and ex-smoker (0–1 year) categories, there were more nonsmokers in the HVA cluster than in the EVA cluster, resulting in a significant distinction (*p* < 0.001) (Fig. 3).

**Fig. 4:**
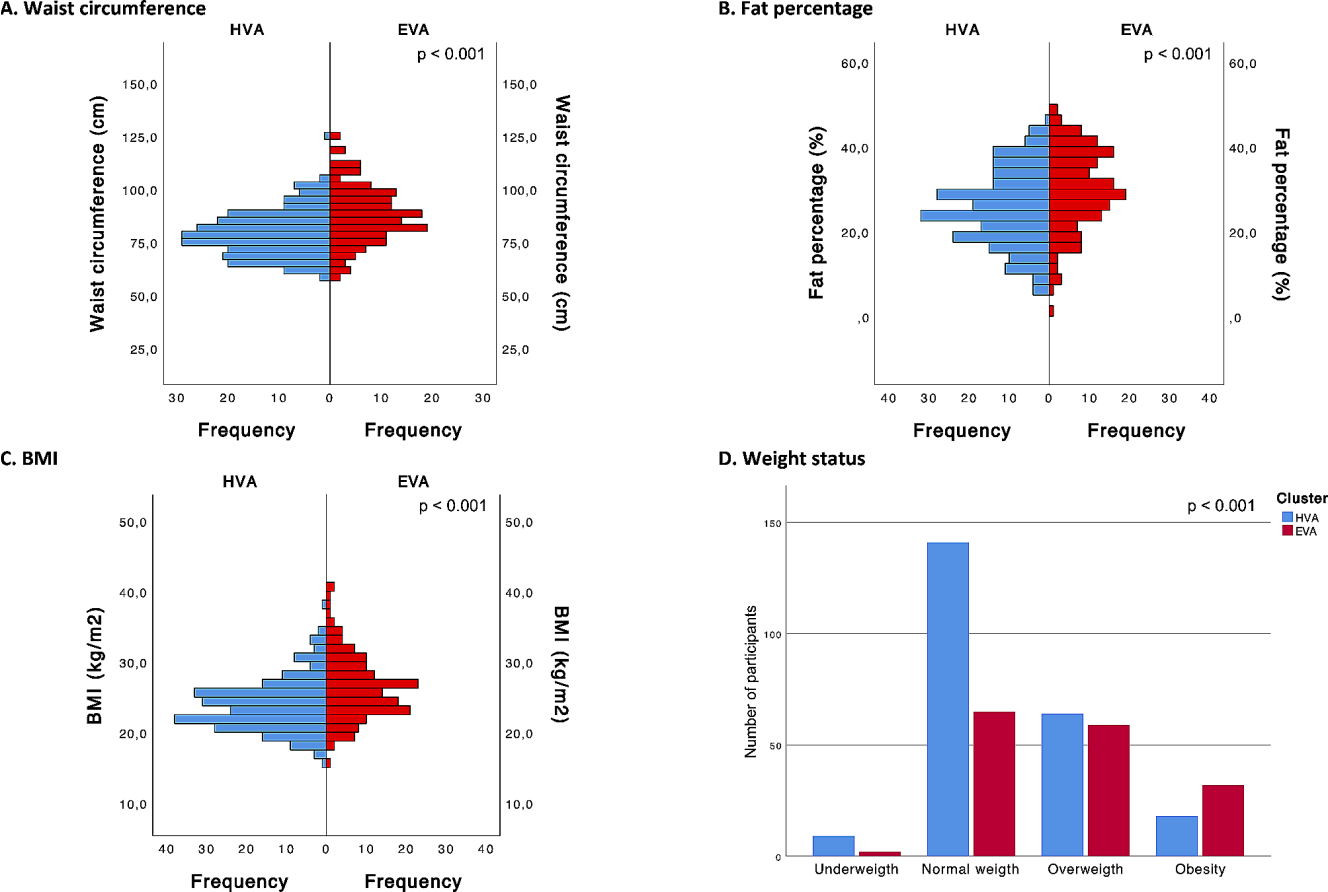
Differences between the cluster categories (HVA and EVA) for adiposity factors For **A**, **B** and **C**, *p* values were estimated using the Mann‒Whitney U test, and for **D**, *p* values were estimated using the chi-squared test. HVA, healthy vascular aging; EVA, early vascular aging; BMI, body mass index

Within the adiposity factors, our analysis revealed that individuals in the EVA cluster had significantly higher BMI values than those in the HVA cluster (*p* < 0.001). Similarly, waist circumference showed a substantial increase in the EVA cluster, emphasizing the role of adiposity in vascular aging (*p* < 0.001). Furthermore, a discernible elevation in fat percentage was observed in the EVA cluster, suggesting the importance of adipose tissue in the early vascular aging process (*p* < 0.001). In terms of weight status, the EVA cluster exhibited a higher prevalence of individuals classified as overweight and obese in comparison to the HVA cluster (*p* < 0.001) (Fig. 4).

**Fig. 5:**
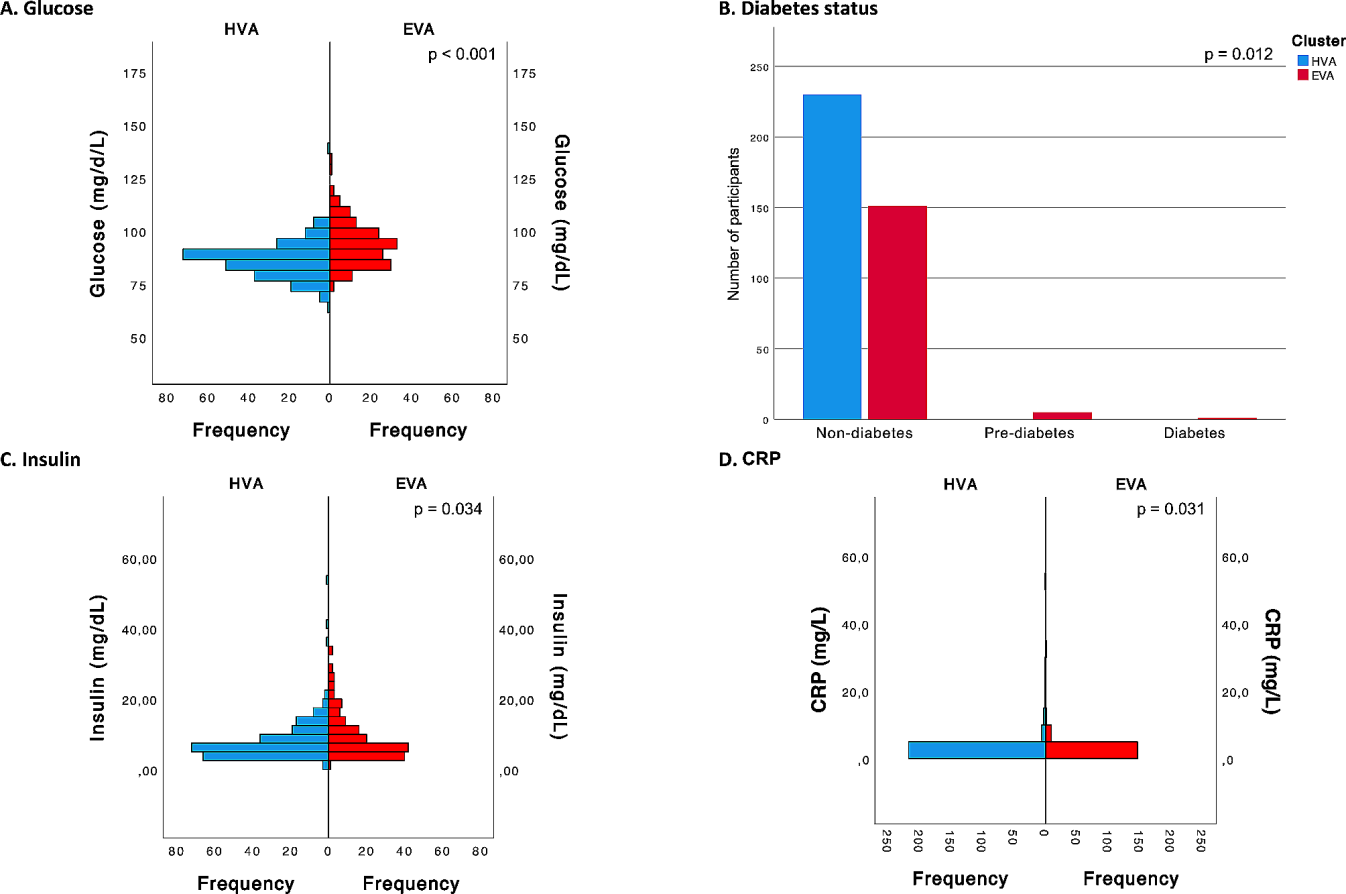
Differences between the cluster categories (HVA and EVA) for glycemic and inflammatory factors For **A**, **C** and **D**, *p* values were estimated using the Mann‒Whitney U test, and for **B**, *p* values were estimated using the chi-squared test. HVA, healthy vascular aging; EVA, early vascular aging; CRP, c-reactive protein

Regarding glycemic and inflammatory factors, our examination showed a significant increase in glucose levels in the EVA cluster compared to the HVA cluster (*p* < 0.001). While insulin levels and CRP showed slight increases in the EVA cluster, these differences were statistically significant (*p* = 0.034 and *p* = 0.031, respectively). Notably, although the majority of individuals in both clusters were nondiabetic, the prevalence of prediabetes and diabetes was slightly higher in the EVA cluster (*p* = 0.012) (Fig. 5).

**Fig. 6:**
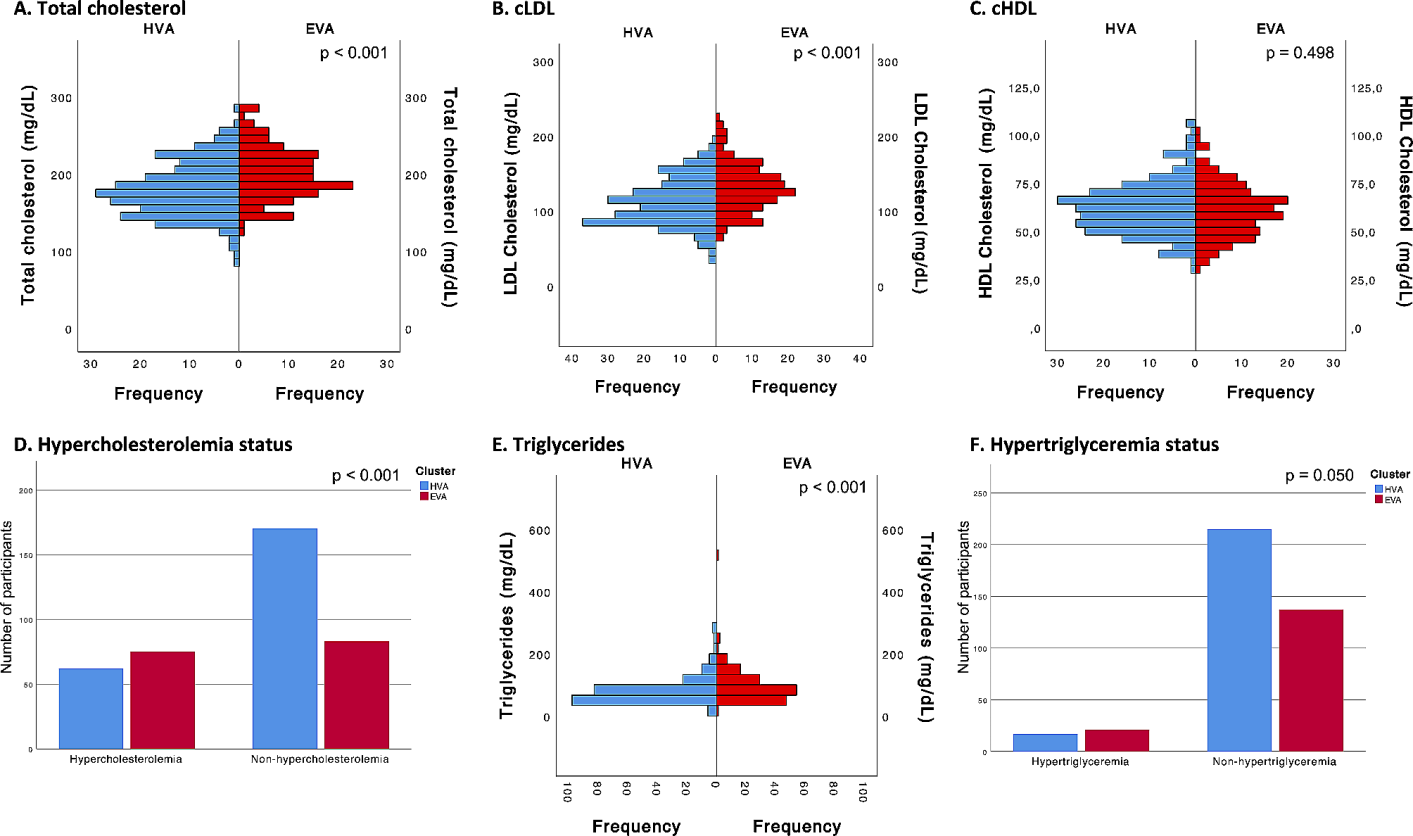
Differences between the cluster categories (HVA and EVA) for lipid profile factors For **A**, **B**, **C** and **E**, *p* values were estimated using the Mann‒Whitney U test, and for **D** and **F**, *p* values were estimated using the chi-squared test. HVA, healthy vascular aging; EVA, early vascular aging; HDL, high-density lipoprotein cholesterol; LDL, low-density lipoprotein cholesterol

In terms of lipid profile factors, our findings highlighted substantial elevations in the levels of total cholesterol, LDL, and triglycerides in the EVA cluster compared to the HVA cluster (*p* < 0.001). In particular, no statistically significant variations in HDL levels were observed between the two clusters. The EVA cluster also had a higher proportion of individuals with hypercholesterolemia than the HVA cluster (*p* < 0.001). Additionally, a modest inclination in hypertriglyceridaemia was observed in the EVA cluster, although it did not reach statistical significance (*p* = 0.051) (Fig. 6).

**Fig. 7:**
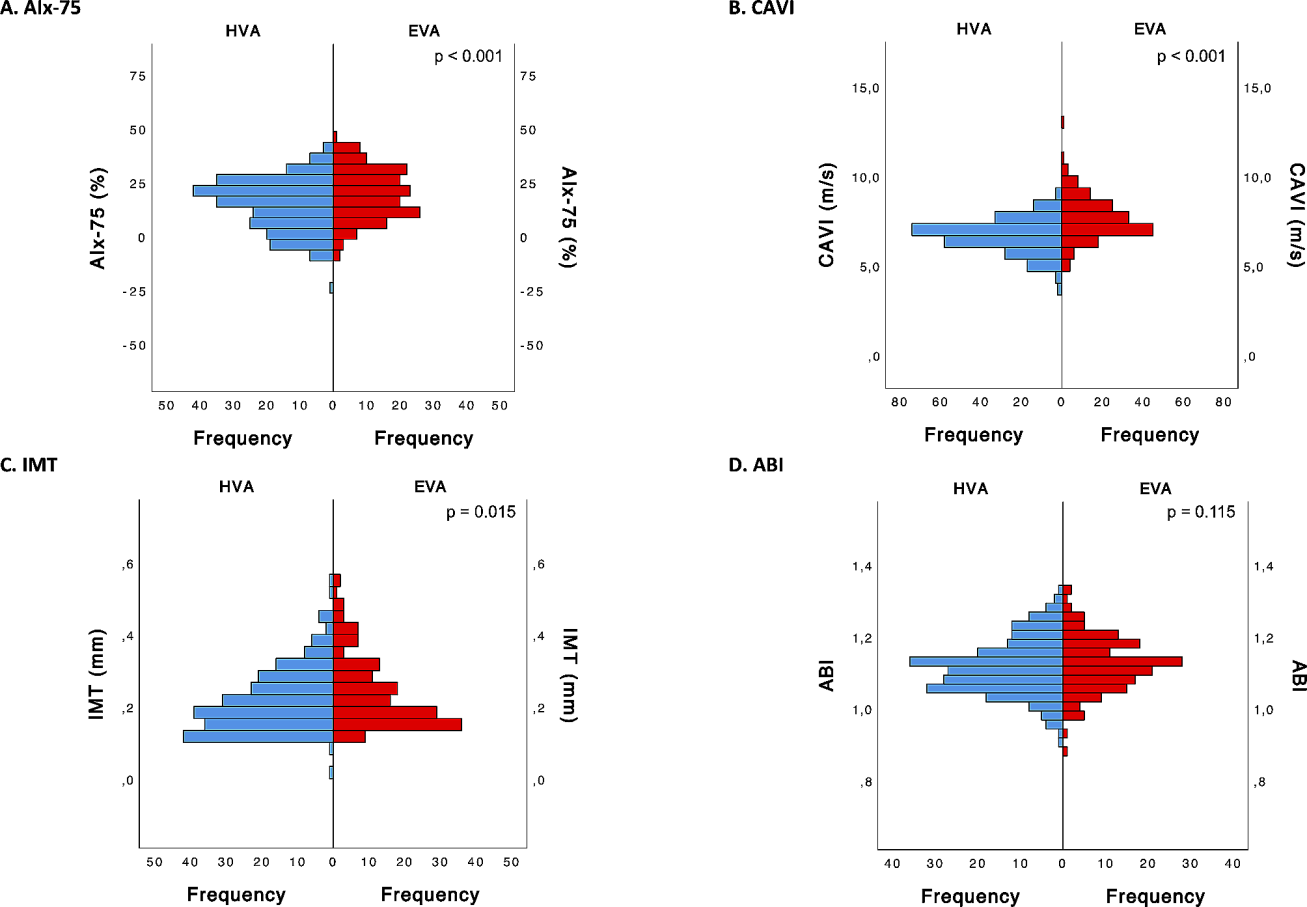
Differences between the cluster categories (HVA and EVA) for vascular factors For **A**, **B**, **C** and **D**, *p* values were estimated using the Mann‒Whitney U test. HVA, healthy vascular aging; EVA, early vascular aging; AIx@75, augmentation index; ABI, ankle-brachial index; CAVI, cardio-ankle vascular index; IMT: intima media thickness

Among the vascular factors, AIx-75 showed a significant increase in the EVA cluster compared to the HVA cluster, suggesting altered arterial stiffness and wave reflection properties in early vascular aging (*p* < 0.001). However, no statistically significant differences in ABI were observed between the two clusters. CAVI showed a significant elevation in the EVA cluster, signifying greater vascular stiffness (*p* < 0.001). Additionally, our analysis revealed that IMT was slightly greater in the EVA cluster, with statistically significant differences (*p* = 0.015) (Fig. 7).

**Fig. 8:**
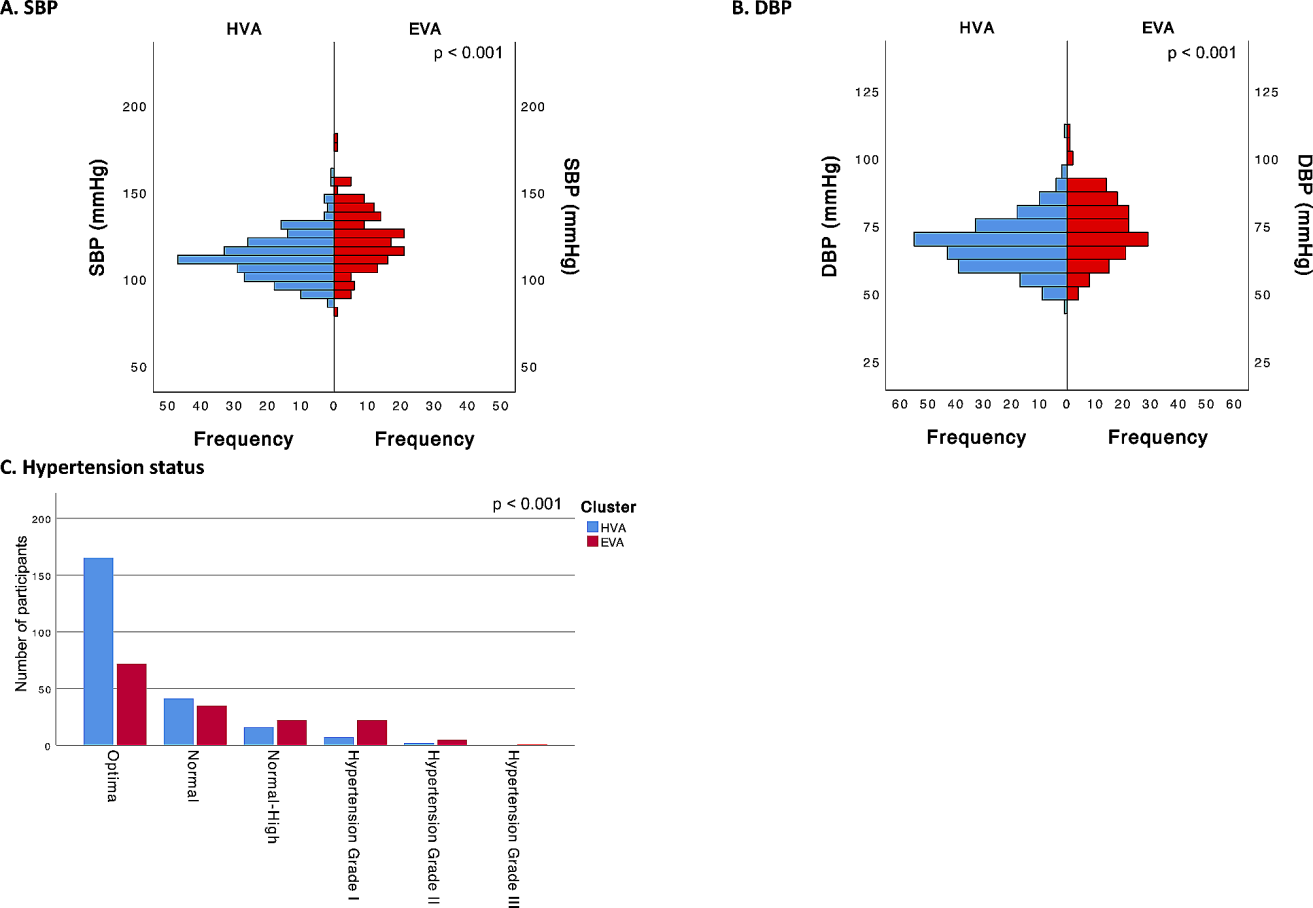
Differences between the cluster categories (HVA and EVA) for blood pressure factors For **A** and **B**, *p* values were estimated using the Mann‒Whitney U test, and for **C**, *p* values were estimated using the chi-squared test. HVA, healthy vascular aging; EVA, early vascular aging; SBP, systolic blood pressure; DBP, diastolic blood pressure

Finally, in terms of blood pressure factors, both SBP and DBP were substantially higher in the EVA cluster than in the HVA cluster (*p* < 0.001). Importantly, our analysis revealed a higher proportion of hypertension, especially in the categories of hypertension grade I and grade II, in the EVA cluster (*p* < 0.001) (Fig. 8).

## Discussion

In summary, our findings provide valuable insights into the potential utility of the clustering model for the assessment of EVA and its associated cardiovascular risk. The results indicate that the clustering model effectively stratifies individuals into two distinct groups, HVA and EVA, based on a set of construct variables. These findings align with the growing recognition of EVA as a key factor in cardiovascular risk assessment. EVA manifests through structural and functional alterations in the vascular system, including arterial stiffness, endothelial dysfunction, and variations in intima-media thickness. Our results confirm that individuals with EVA exhibit significant differences in various risk factors, including age, adiposity, glycemic control, lipid profiles, vascular health, and blood pressure, compared to those in the HVA group.

Age is a well-established risk factor for cardiovascular disease, but its specific role in EVA has been a subject of interest in recent research [4]. In this study, individuals in the EVA cluster were significantly older than those in the HVA group. This finding aligns with the concept that EVA is a process characterized by accelerated vascular aging [32]. As individuals age, a number of structural and functional changes occur in the vascular system, including increased arterial stiffness and endothelial dysfunction [7]. EVA may represent an extreme manifestation of these age-related changes. Early detection and intervention in older populations is crucial because these individuals may already be in an advanced stage of vascular aging, which may result in an increased risk of cardiovascular events [33]. This finding underscores the need for age-specific interventions and risk assessments in clinical practice [34].

The significantly higher levels of adiposity factors, including BMI, waist circumference, and fat percentage, in the EVA group suggest a strong association between adipose tissue and early vascular aging. Adipose tissue is known to produce various bioactive substances, such as cytokines and adipokines, which can contribute to inflammation and endothelial dysfunction [35]. Moreover, adiposity is often associated with other risk factors, such as insulin resistance, dyslipidemia, and hypertension, all of which are known to accelerate vascular aging [36]. Therefore, addressing weight management and reducing adiposity may be crucial in EVA prevention. Lifestyle modifications, including dietary changes and regular physical activity, may play a significant role in mitigating the adverse effects of adiposity on vascular health [37].

The elevated glucose levels and the observed higher prevalence of prediabetes and diabetes in the EVA group suggest a strong link between glycemic control and EVA. Chronic hyperglycemia is known to induce oxidative stress and inflammation, which can contribute to endothelial dysfunction and vascular damage [38]. Moreover, the inclusion of HbA1c in the construct is primarily based on the analysis of the EVA construct previously analysed [9]. HbA1c in apparently healthy individuals is crucial because of its ability to provide an integrative measure of long-term glycaemic control [39]. Since chronic hyperglycaemia is a known risk factor for the development and progression of cardiovascular disease [40], the inclusion of HbA1c in the EVA construct may more accurately identify those individuals who, despite having apparently normal fasting glucose values, may experience detrimental fluctuations in their glucose levels over time. Consequently, integrating HbA1c in apparently healthy subjects may improve the predictive ability of cardiovascular risk by more effectively capturing chronic glycaemic load, thus contributing to more personalised and effective preventive strategies [41].

Similarly, the elevated lipid profiles (total cholesterol, LDL, and triglycerides) in the EVA group highlight the significance of lipid management in EVA prevention. Dyslipidemia can lead to atherosclerosis and increased vascular stiffness, contributing to early vascular aging [42]. The findings related to vascular factors (arterial stiffness and IMT) reinforce the importance of assessing vascular health for the early detection and management of EVA. The higher prevalence of hypertension in the EVA cluster underlines the role of blood pressure management in EVA risk reduction. Elevated blood pressure can lead to structural changes in blood vessels and increased vascular resistance, further accelerating vascular aging [43].

The external validation of the clustering model is crucial for several reasons. First, it confirms that the model’s clusters align with established risk factors. This strengthens the model’s clinical utility and suggests that it can identify individuals at risk of cardiovascular events based on readily available parameters. Second, the external validation underscores the importance of these traditional risk factors in EVA. This suggests that EVA is not an isolated phenomenon but rather a manifestation of cardiovascular risk factors that can be identified and managed using established guidelines. This, in turn, highlights the potential for early intervention in individuals showing signs of EVA.

However, important limitations should be acknowledged for this study. First, the size of the study population, deemed too small, may impact the statistical power and generalizability of the findings. Additionally, the age composition, primarily comprising young to middle-age subjects with a low rate of cardiovascular events in the general population, introduces a potential limitation in extrapolating the results to broader age groups with different cardiovascular risk profiles. The cross-sectional design of the research restricts our ability to test the proposed hypothesis thoroughly. Longitudinal studies are imperative to elucidate causal relationships and comprehensively assess the impact of identified risk factors on the development of early vascular aging (EVA) over time. Additionally, the study’s findings are based on a specific population, and their generalizability to other demographic groups should be investigated. Furthermore, the clustering model’s performance may vary in different clinical and research settings, and its robustness needs further validation in diverse populations.

In conclusion, the results of this study support the validity and utility of the clustering model for assessing EVA and its association with cardiovascular risk. The differentiation between the HVA and EVA groups based on various risk factors provides a comprehensive understanding of EVA’s distinct characteristics. These findings have implications for clinical practice, as they suggest that interventions targeting age, adiposity, glycemic control, lipid profile, vascular health, and blood pressure may be particularly relevant for individuals at risk of EVA. Further research and longitudinal studies are needed to confirm these associations and to assess the long-term impact of EVA on cardiovascular outcomes. Successful validation of this clustering model could confirm its utility for model integration into clinical settings, offering healthcare professionals a robust tool for early and accurate cardiovascular risk assessment and management.

## Data Availability

All data generated or analysed during this study are included in this published article.

## Abbreviations

ABI: Ankle-brachial index
ACC: American College of Cardiology
ADA: American Diabetes Association
AGEs: Advanced glycation end products
AHA: American Heart Association
Aix-75: Augmentation index
BMI: Body mass index
CAVI: Cardio ankle vascular index
CRP: Ultrasensitive C-reactive protein
CVD: Cardiovascular disease
DBP: Diastolic blood pressure
EVA: Early vascular aging
HDL: High-density lipoprotein cholesterol
HbA1c: Glycated hemoglobin A1c
HVA: Healthy vascular aging
IMT: Intima media thickness
IQR: Interquartile range
LDL: Low-density lipoprotein cholesterol
PCA: Principal component analysis
PCR: Protein c-reactive
PP: Pulse pressure
PWv: Pulse wave velocity
SAF: Skin autofluorescence
SBP: Systolic blood pressure
STROBE: Strengthening the Reporting of Observational Studies in Epidemiology
